# Thoracic Endovascular Aortic Repair (TEVAR) for Grade III Traumatic Thoracic Aortic Injury

**DOI:** 10.7759/cureus.86454

**Published:** 2025-06-20

**Authors:** Paola Andrea Rojas Guevara, Elias Gallardo-Navarro, Luis Alberto Garnica León, Gisela Margarita Vargas Mendez, Héctor Segura Marín, Venancio Pérez Damian

**Affiliations:** 1 Angiology, Vascular and Endovascular Surgery, Hospital Español, Mexico City, MEX; 2 General Surgery, Hospital Español, Mexico City, MEX

**Keywords:** blunt thoracic trauma, endovascular surgical repair, general and vascular surgery, tevar complications, thoracic aorta

## Abstract

The thoracic endovascular aortic repair (TEVAR) procedure has become a preferred option for managing traumatic lesions of the thoracic aorta due to its ability to intervene in a minimally invasive manner and reduce the risk of complications. This approach allows rapid patient stabilization by avoiding open surgery and facilitates the management of critical conditions, such as pseudoaneurysms of the descending aorta. Aortic contusion injury caused by trauma and deceleration is an important and serious cause of mortality related to trauma, since most of those who suffer it die at the site of the accident or in the first hours of hospital care. Hence, the clinical suspicion and the rapid approach during primary care when locating serious conditions, such as thoracic aortic injury, are of utmost importance for the survival of the affected patient. A 26-year-old woman who suffered a thoracic aortic injury due to automobile trauma was brought to the ED. She underwent TEVAR with endoprosthesis placement due to a diagnosis of pseudoaneurysm and dissection of the descending thoracic aorta (aortic isthmus). The endovascular repair was adequate. The follow-up was conducted with thoracic angiotomography, also known as a CT angiography of the chest, a medical imaging procedure that uses X-rays and a contrast dye to visualise the blood vessels in the chest, particularly the thoracic aorta, at the time of the procedure and 12 months postoperatively. The patient's clinical evolution was favourable, with the endovascular intervention proving to be an adequate option for both diagnostic and therapeutic emergency management.

## Introduction

Thoracic aortic trauma injury occurs most frequently as a sequela of high-impact deceleration, regularly occurring in high-speed traffic accidents and falls from great heights. It is the second leading cause of mortality related to these events, surpassed only by fall-related death from traumatic brain injury [1]. About 90% of thoracic aortic trauma victims die at the site of trauma, and the remainder die within 24 hours, often with multiple associated injuries, including cardiac injuries, multiple rib fractures, hemothorax, and intra-abdominal injuries [1-3]. Most traumatic aortic injuries occur in younger people who have been involved in high-speed motor vehicle collisions, are described in head-on collisions occurring in 60% and are more common than side-impact collisions at 21%; these accidents are followed by people hit by motor vehicles, motorcycle collisions and a fall from a significant height, in decreasing order of frequency [2-4]. The anatomical sites, also known as transition sites, are segments that include the aortic root, the aortic isthmus, and the diaphragmatic aortic hiatus. The ligamentum arteriosum is immobile, so shearing from trauma produces a closed injury of the thoracic aorta, which is caused by high-velocity forces [5,6]. The portion of the descending thoracic aorta is the site most commonly affected by an aortic lesion, described as a subcentimetric anomaly of the intima, which can form a hematoma or produce a flap without generating deformity of the external contour. This lesion is involved in two-thirds of patients presenting to the ED. The lesions are also described in smaller proportions, with incidences of approximately 8% to 27% in the ascending aorta, 8% to 18% in the aortic arch, 11% to 21% in the distal descending aorta, and 7% to 22% in the abdominal aorta [6,7]. Understanding and suspecting the mechanics of trauma makes diagnosis easier in terms of staging and treatment of aortic injuries. CT scan covers a wide range of lesions, from small intraluminal defects to full-thickness circumferential ruptures [2,8]. CT findings include intimal tear or localized hematoma (Grade I), pseudoaneurysm affecting less than 50% of the total aortic circumference (Grade II), pseudoaneurysm affecting more than 50% of the total aortic circumference (Grade III), and contrast extravasation or rupture (Grade IV). Other findings that can be observed include periaortic hematoma, hemothorax, and traumatic injury to the thoracic aorta; these findings should be recognized and addressed as soon as possible to improve the survival rate in these patients [6,8,9]. Treatment strategies for closed thoracic injuries currently coincide with the development of a new staging system and the use of thoracic endovascular aortic repair (TEVAR) as a rapidly evolving therapy in the treatment of various thoracic aortic pathologies in trauma [3,7,10].

## Case presentation

A 26-year-old woman with no significant medical history presented to the emergency room two hours after suffering an automobile accident where she was driving, with several contusions, cranioencephalic trauma, retrograde amnesia, retrosternal thoracic pain radiating to the back, left forearm, cervical spine, right inguinal region, and left knee, suffering fractures in multiple bilateral costal levels, exposed fracture of the right tibia and ipsilateral linear fracture of the femur. Upon arrival at the hospital, the fractures were stabilized with external fixators. A polytrauma protocol was initiated, involving multidisciplinary management with the services of traumatology, orthopaedics, thoracic surgery, neurosurgery, angiology, and vascular surgery. On physical examination, the patient was conscious, oriented, and cooperative. Skull without exostoses or collapse, isochoric pupils, central trachea, thorax with pain on deep inspiration and palpation of the left upper hemithorax and at the level of the right fourth arch, abdomen not painful on palpation, with no evidence of peritoneal irritation. Her vital signs were within normal parameters.

**Figure 1:**
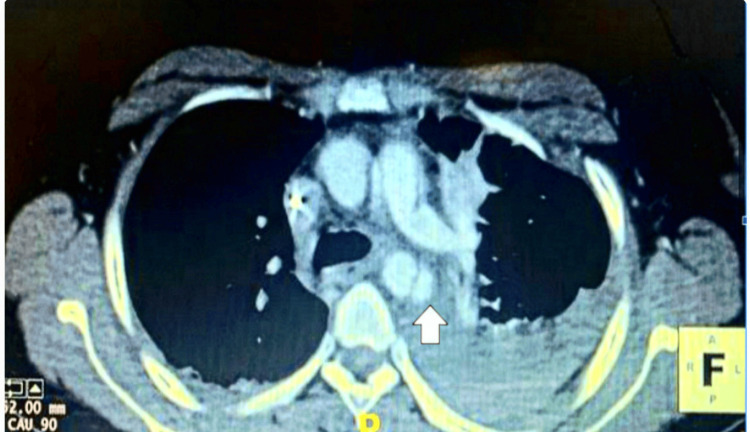
Computed tomography of axial section of the thorax (arrow) shows the presence of aortic dissection of the descending aorta.

Admission laboratory tests revealed the following results: myoglobin 668.8 ng/mL (normal range: 25-72 ng/mL), creatinine 1.51 mg/dL (normal range: 0.7-1.3 mg/dL), international normalized ratio 1.05 (normal range: 0.8-1.2), leukocytes 14.1x10^9^/L (normal range: 4.5-11.0x10^9^/L), haemoglobin 12.1 g/dL (normal range: 11.6-15 g/dL), platelets: 244x10^9^/L (normal range: 150-400x10^9^/L), creatine phosphokinase (CPK) 2870 U/L (normal range: 29-200 U/L), CPK-MB 51.8 U/L (normal range: <25 U/L), and troponin I 151.2 ng/mL (normal range: <0.04 ng/mL). A chest X-ray revealed fractures from the fourth to the seventh right costal arch and from the second to the seventh left costal arch. A chest angiotomography (Figure 1) confirmed these fractures and additionally showed a left hemothorax, an intramural hematoma in the descending aorta measuring up to 2.3 cm, an intimal flap, and a pseudoaneurysm.

**Figure 2:**
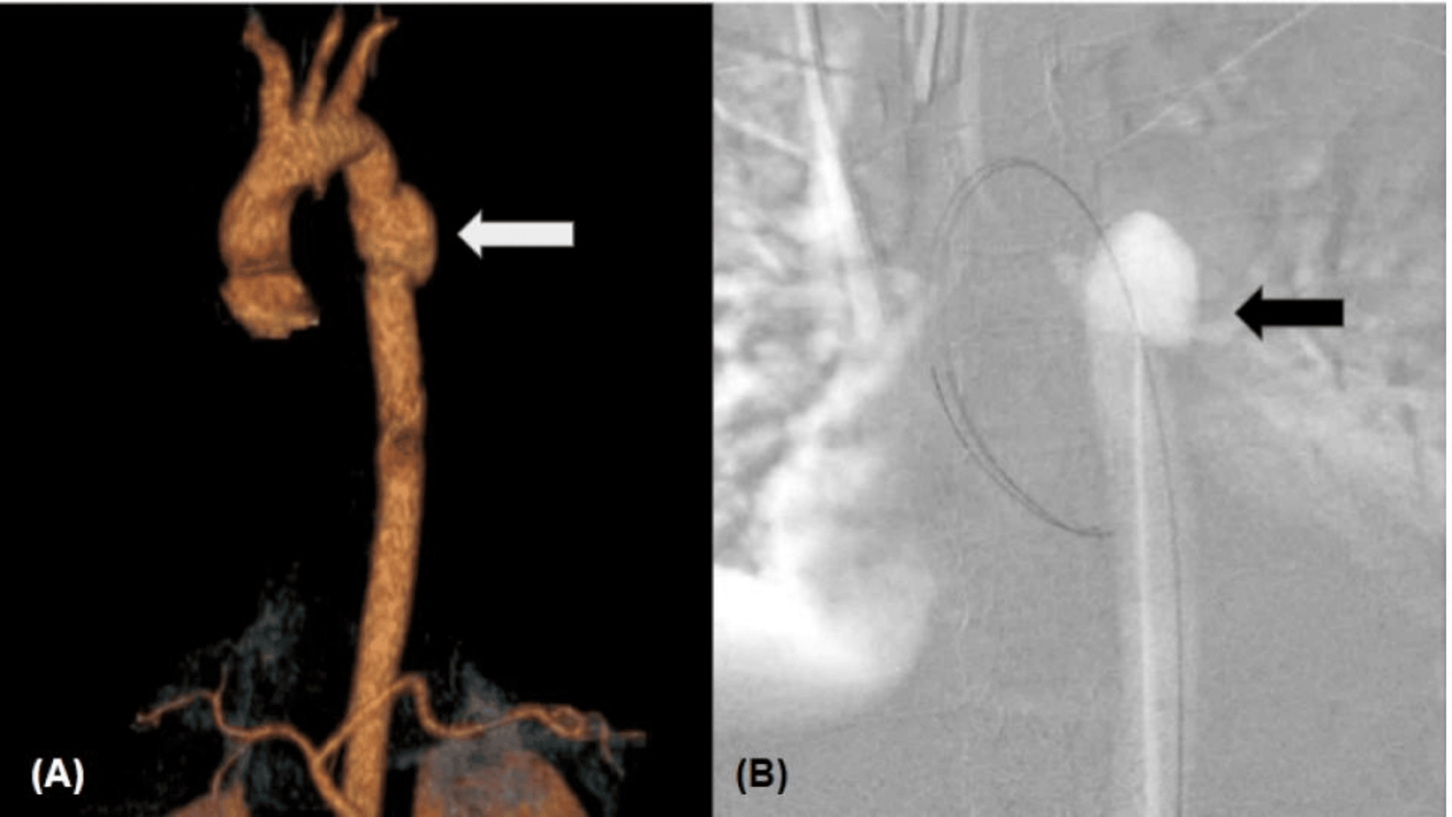
(A) Showing a pseudoaneurysm of the descending thoracic aorta originating immediately after the emergence of the left subclavian artery. (B) Showing a pseudoaneurysm of the descending aorta without evidence of contrast medium leakage.

**Figure 3:**
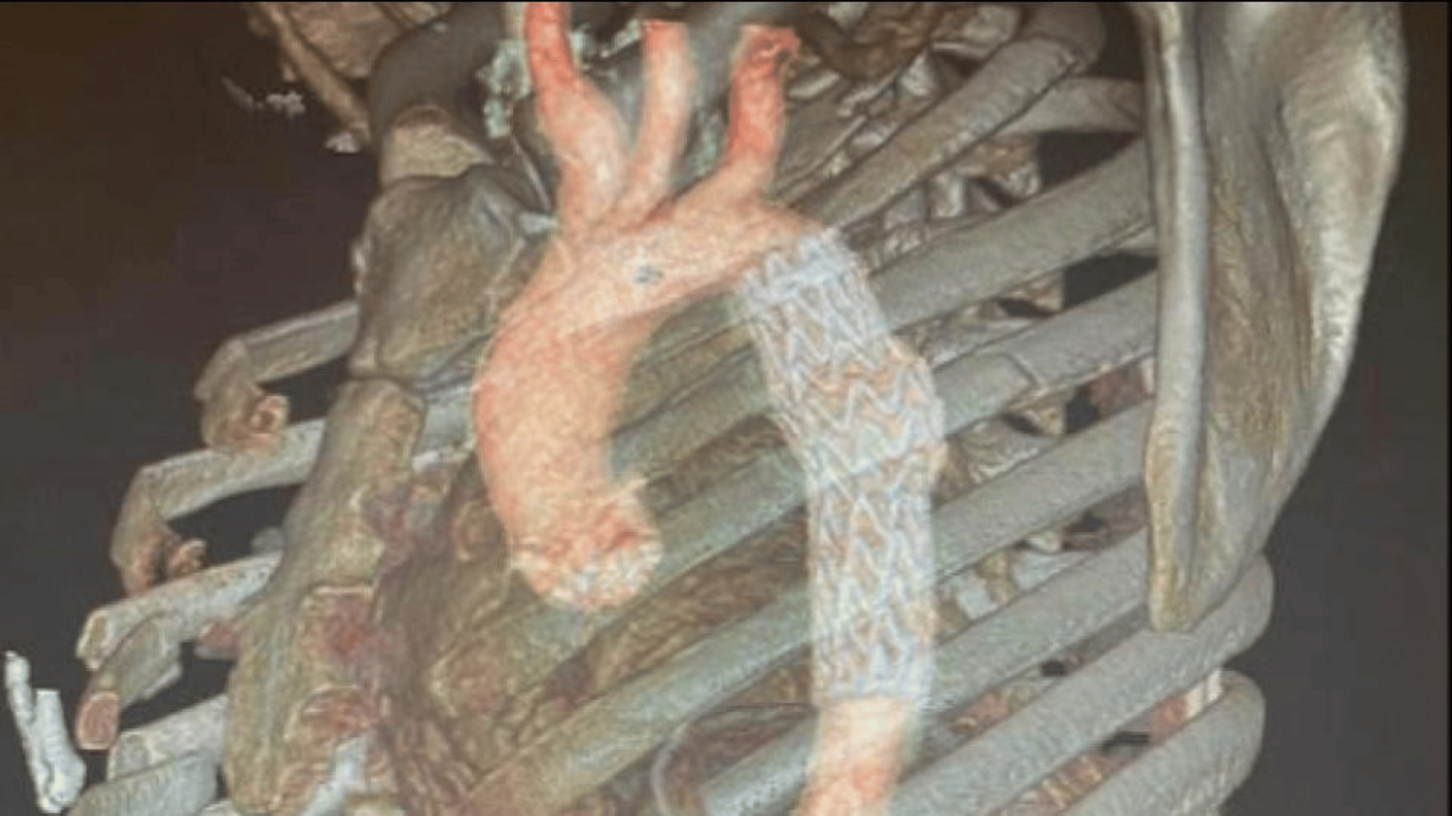
Sagittal view of chest angiotomography with 3D reconstruction following treatment

Therefore, a surgical plan was decided by the angiology and vascular surgery service, which consisted of aortography and placement of a thoracic aortic (GORE-TEX® Vascular Grafts) 26-26 x 100 mm endoprosthesis (Figure 2) through vascular accesses of the brachial and right femoral artery with a 6 Fr pigtail catheter, with a diagnosis of thoracic aortic trauma grade III with pseudoaneurysm. In the ICU, she continued with thoracic surgery management, during which two more surgical interventions were performed: left hemothorax aspiration with placement of a 32 Fr endopleural tube and fracture reduction with osteosynthesis of the costal arches. In the post-surgical follow-up, a chest angiotomography with 3D reconstruction (Figure 3) was performed, where adequate placement of the endoprosthesis was observed, without migration or endoleak. The patient was discharged after three weeks of management, with no comorbidities affecting her quality of life.

**Figure 4:**
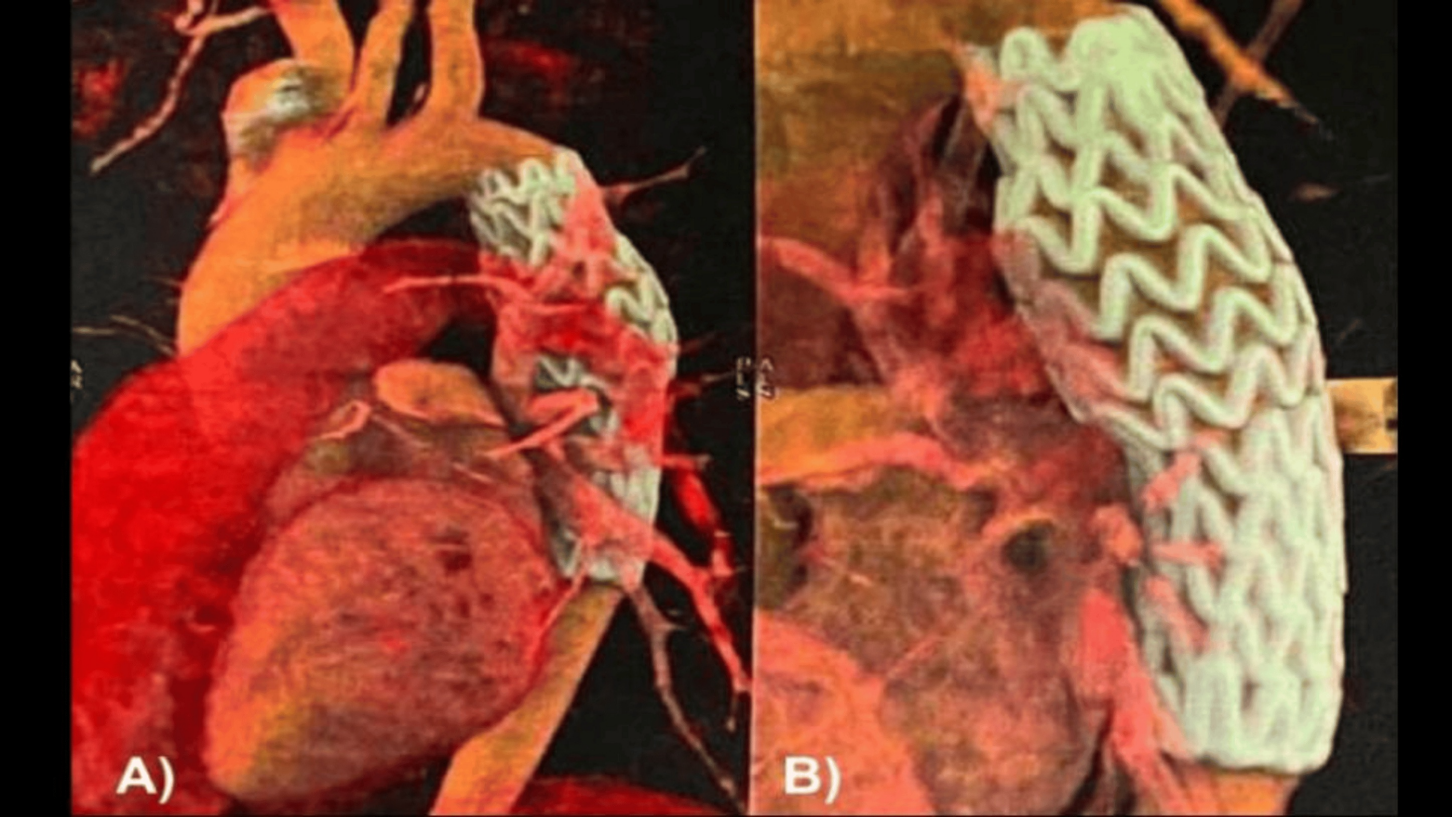
(A-B) Sagittal section and 3D reconstruction of chest angiotomography at 12 months post-treatment

She was seen in consultation without complications, and angiotomography was requested 12 months after treatment, revealing the adequate position of the endoprosthesis without evidence of migration or leakage (Figure 4).

## Discussion

Acute aortic syndrome encompasses three diseases that represent a group of patients with similar symptoms, including penetrating ulcer, intramural hematoma, and aortic dissection. Some series integrate incomplete aortic dissection by a laceration of the aortic wall without involvement of the middle layer [11-13]. The first description of traumatic rupture of the aorta was made in 1557 by Vesalius, who observed a patient with an aortic rupture after a fall from a horse. Later, in 1923, Dshanelidze in Russia described the first successful repair of a traumatic aortic rupture resulting from a penetrating injury. In the 1950s, more reports were added by Gerbode, Klassen, Passaro, and Pace [14-16].

The American Association for the Surgery of Trauma classified thoracic vascular lesions according to the type of artery and the extent of arterial circumference involved. In relation to this classification, all lesions of the descending thoracic aorta were classified as Grade IV [3,15,17]. This classification did not account for the heterogeneity of injuries in the thoracic aorta; however, the system of classification and treatment of traumatic aortic injuries published in 2024 improves the description of aortic injury, taking into account the natural history of different types of injuries. This classification system is based on the anatomical layers of the aortic wall and includes the following grades: Grade I: intimal tear, medical treatment is recommended (Class IIa), Grade II: intramural hematoma, medical treatment/TEVAR is recommended (Class IIa), Grade III: pseudoaneurysm, treatment with TEVAR/open surgical repair is recommended (Class I), and Grade IV: rupture, emergency treatment with TEVAR/open surgical repair is indicated (Class I). It also agrees with the Adams classification, which describes the type of thoracic aortic injury due to trauma in Grade I (intimal tear), Grade II (intramural hematoma), Grade III (pseudoaneurysm), and Grade IV (rupture) [3,15,18].

The thoracic aorta is joined by multiple vascular and non-vascular structures, such as the branches of the aortic arch, the ligamentum arteriosum, the heart, the intercostal arteries, and the diaphragmatic pillars. Transitional sites between the mobile and fixed segments are known to include the aortic root, aortic isthmus, and diaphragmatic aortic hiatus [2,17,19]. Current general theory suggests that the convergence of multiple simultaneous injury forces can cause synchronous injury in the thoracic aorta. In addition, a displaced vertebral fracture can cause direct trauma to the aorta along with shear force, which has been shown to play a less important role in abdominal aortic injury than in thoracic injury by this type of mechanism [2,18,20]. Diagnosis begins with a thorough history and a comprehensive physical examination. The initial assessment conforms to each hospital's advanced life support trauma guidelines. At the initial stage, symptoms may vary; they can include clinical signs of hypovolemic shock and chest pain radiating to the back. Consider this type of injury if bilateral rib fractures are present at multiple levels [3,13,16].

Significant findings of the physical examination include distension of the neck veins, absent cardiac noises, tracheal deviation, subcutaneous emphysema, chest wall instability or ecchymosis, abnormal respiratory noises, and decreased peripheral pulses [2,17,19]. Initial management includes primary and secondary examinations, as well as complementary tests that are appropriate to assess the patient's clinical status. In cases of suspected chest aortic trauma, blood pressure control and pain management are also employed to prevent the progression of injury by reducing stress on the aortic wall [15,18,20]. The risk of rupture has been shown to decrease from 1.5% to 12% with effective antihypertensive therapy. Optimal hemodynamic parameters are not well established, but systolic blood pressure below 100 mmHg is recommended, mean blood pressure less than 80 mmHg and heart rate less than 100 beats per minute, usually used with medications such as an intravenous beta-blocker such as esmolol, labetalol and may be supplemented with a vasodilator such as nitroprusside [3,18,21]. It is essential to note that pharmacological control of blood pressure and heart rate directly reduces the risk of aortic rupture.

In the emergency department, the initial imaging modality is a chest X-ray, which detects radiographic findings suggestive of aortic rupture, including an enlarged mediastinum, left pleural effusion, fractures of the first and second ribs, tracheal deviation, a depressed left bronchus, an indistinct aortic button, or an apical coating [3,13]. Although angiography was considered the gold standard for diagnosing closed aortic lesions for nearly four decades, thoracic angiotomography is now considered the diagnostic test of choice [3,13,20]. Therefore, the use of angiotomography is crucial in both diagnosis and treatment planning, including the evaluation of endovascular stents.

The clinical practice guidelines of the Society of Vascular Surgery recommended an urgent TEVAR for Grade II-IV by placing endoprosthesis to cover the injury of the intima and allow a heart rate and blood pressure to be sufficient to prevent strokes and paraplegia, with a TEVAR complication rate in the general management of traumatic aortic injury of 3%-18%, these lesions are used antihypertensive therapy, the division of treatment to be done in less than 24 hours, for patients in an unstable hemodynamic condition with aortic lesions that are observed with external contour alterations such as pseudoaneurysm, complete aortic transection, grade III and IV aortic lesion, emergency treatment is recommended. For Grade I or II lesions, treatment may be given after 24 hours or after 15 days, depending on the severity of concomitant lesions. Additionally, conservative management includes cases of intima lesions >1 cm and small pseudoaneurysms <1 cm, sometimes having spontaneous healing within the first 4-8 weeks, with only 10%-15% of cases persisting or progressing during the follow-up interval, with favourable results [14,18,20].

The endovascular approach is dependent on the area of the aorta; as a rule, it should be 2 cm proximal and 10 cm distal of the lesion. This is assessed before surgical intervention using a 3D reconstruction with angiotomography, taking appropriate diameter measurements at these two sites. As an alternative to this imaging study, intravascular ultrasound should be used to ensure the size of the graft is chosen [2,13,17,21]. The requirements for access vessels depend on the device, but the typical limiting factor is iliac arteries with a diameter of 7 mm, 10 to 20% over-dimensioning is recommended when placing the stent, but with a tendency to remain below that range, most endovascular complications can be treated by minimally invasive means, without requiring open surgery [15,18,19]. A variety of retrospective series of patients with traumatic aortic lesions treated with TEVAR have shown successful results, as in a study conducted at the Arizona Heart Institute, which implanted this type of stent in 158 patients, with several thoracic aortic lesions, some of which were at high risk, and the following complication rates were observed, transient paraparesis (1.9%), paraplegia (0.6%) and endoleakage (11.5%), with a 30-day mortality rate of 3.8% and at 21 months total mortality was 17.3% [13,14].

The Society of Thoracic Surgeons/American Association for Thoracic Surgery (STS/AATS) guidelines, published in 2022, propose that prophylactic TEVAR should also be considered in patients with adequate anatomy and high-risk characteristics [16,18,19]. TEVAR is reserved for patients without an adequate response to medical therapy; this approach was supported by the initial results of the INvestigation of STENT grafts in patients with acute type B Aortic Dissection (INSTEAD) trial, which compared medical management to TEVAR and found no difference in all-cause mortality at 1 year [22]. However, open repair remains an initial treatment option at times, especially when a patient's lesion has anatomical characteristics that are not suitable for an endovascular approach, such as the type of aortic arch, curvature, diameter of the proximal support area, and characteristics of the access iliac vessel. Currently, although open repairs have more lasting results and require fewer interventions, the risk of early complications is significantly higher than endovascular intervention [14,17,18].

To prevent complications, the size of the vascular graft should be evaluated beforehand, as it can cause migration, graft infection, pseudocoarctation, occlusion and endofuga, the last being defined as the persistence of blood flow outside the graft but inside aneurysmal sac, is the most common complication, whose incidence reaches up to a third of procedures and manifests itself in the short or long term, regularly after the first year [15,18,21]. The serious complications in both procedures are stroke and paraplegia, but the rare complication of TEVAR is spinal cord injury. Early complications are more common in open surgical repair, but late complications have been reported with greater incidence in endovascular repair [16,18,21].

## Conclusions

Mortality is high due to this type of injury, but medical care, timely diagnosis, and endovascular therapy have improved outcomes for these patients. Endovascular therapy for treating traumatic aortic lesions has introduced new challenges. To investigate changes in the long-term remodeling of the thoracic aorta after stent placement in young patients, we observed no complications during the year of follow-up, which is a suitable form of emergency treatment for traumatic injuries. Adequate seal zones, careful preoperative planning, and proper device sizing are crucial for achieving favorable results and minimizing complications. Hybrid approach options and fenestrated endografts may further expand the number of patients who can benefit from this treatment modality.
